# Supplementary material for: Mouse Pachytene piRNAs Cleave Hundreds of Transcripts, But Alter the Steady-State Abundance of Only a Minority of Targets
Source: bioRxiv. 2024 Nov 3:2024.11.02.621675. Preprint. [Version 1] doi: 10.1101/2024.11.02.621675 (PMC11566022; doi:10.1101/2024.11.02.621675)

bioRxiv preprint doi: https://doi.org/10.1101/2024.11.02.621675; this version posted November 3, 2024. The copyright holder for this preprint (which was not certified by peer review) is the author/funder, who has granted bioRxiv a license to display the preprint in perpetuity. It is made available under aCC-BY 4.0 International license.

SUPPLEMENTARY FIGURE LEGENDS

Figure S1. Fertility Defects Observed in Male Mice Lacking Two or Three Pachytene piRNA Loci, Related to Figure 1.

(A) Number of viable pups produced by the control (C57BL/6) and pachytene piRNA mutant males in successive matings with C57BL/6 females over 8 months. Median and IQR are shown. Kruskal-Wallis test (one-way ANOVA on ranks) p-value = 2×10^{-7}. Benjamini-Hochberg corrected p-values for *post hoc* pairwise Mann-Whitney tests are shown.

(B) Fraction of motile sperm from caudal epididymis of C57BL/6, $pi9^{-/-}$, $pi17^{-/-}$, and $pi9^{-/-}pi17^{-/-}$ males determined by CASAnova. Kruskal-Wallis test (one-way ANOVA on ranks) p-value = 0.00036. Benjamini-Hochberg corrected p-values for *post hoc* pairwise Mann-Whitney tests are shown.

(C) Fraction of progressive sperm from caudal epididymis of C57BL/6, $pi9^{-/-}$, $pi17^{-/-}$, and $pi9^{-/-}pi17^{-/-}$ males determined by CASAnova. Kruskal-Wallis test (one-way ANOVA on ranks) p-value = 0.000048. Benjamini-Hochberg corrected p-values for *post hoc* pairwise Mann-Whitney tests are shown.

(D) C57BL/6 and $pi9^{-/-}pi17^{-/-}$ sperm function analyzed by IVF using C57BL/6 oocytes with or without zona pellucida.

Figure S2. Steady-State Levels of Putative Cleavage Products Generated by *pi9* and *pi17* piRNA-Guided Slicing, Related to Figure 2.

(A) At left, abundance in C57BL/6, $pi9^{-/-}$, $pi17^{-/-}$, and $pi9^{-/-}pi17^{-/-}$ primary spermatocytes of putative 5′-monophosphate-bearing cleavage products derived from targets of *pi9* piRNAs (shown in Figure 2A). At right, Kruskal-Wallis test p-values (one-way ANOVA on ranks) and Benjamini-Hochberg-corrected p-values for *post hoc* pairwise Mann-Whitney tests are shown for each gene.

bioRxiv preprint doi: https://doi.org/10.1101/2024.11.02.621675; this version posted November 3, 2024. The copyright holder for this preprint (which was not certified by peer review) is the author/funder, who has granted bioRxiv a license to display the preprint in perpetuity. It is made available under aCC-BY 4.0 International license.

(B) At left, abundance in C57BL/6, *pi9*$^{-/-}$, *pi17*$^{-/-}$, and *pi9*$^{-/-}$*pi17*$^{-/-}$ primary spermatocytes of putative 5′-monophosphate-bearing cleavage products derived from targets of *pi17* piRNAs (shown in Figure 2A). At right, Kruskal-Wallis test *p*-values (one-way ANOVA on ranks) and Benjamini-Hochberg-corrected *p*-values for *post hoc* pairwise Mann-Whitney tests are shown for each gene.

Figure S3. Steady-State Levels of Putative Cleavage Products Generated by *pi9* and *pi17* piRNA-Guided Slicing, Related to Figure 4.

(A) Change in steady-state abundance in *pi9*$^{-/-}$*pi17*$^{-/-}$ vs C57BL/6 secondary spermatocytes, round spermatids, and elongating spermatids for mRNAs whose 3′UTRs pair to *pi9* and *pi17* piRNAs or to control piRNAs (i.e. piRNAs whose abundance does not change in *pi9*$^{-/-}$*pi17*$^{-/-}$ primary spermatocytes). Two-tailed KS test *p*-values are shown.
(B) Steady-state levels of Grk4 mRNA in C57BL/6, *pi9*$^{em1/em1}$*pi17*$^{em1/em1}$, and *pi9*$^{em2/em2}$*pi17*$^{em2/em2}$ primary spermatocytes, secondary spermatocytes, round spermatids, and elongating spermatids. Benjamini-Hochberg-corrected *p*-values for Wald test calculated by DESeq2 are shown for each gene.

Figure S4. Steady-State Levels of Putative Cleavage Products Generated by *pi6* piRNA-Guided Slicing, Related to Figure 4.

Abundance in C57BL/6 and *pi6*$^{-/-}$ primary spermatocytes of putative 5′-monophosphate-bearing cleavage products derived from targets of *pi6* piRNAs (shown in Figure 4B). Unpaired, two-tailed Mann-Whitney test *p*-values are shown.

Figure S5. Gene Ontology Terms Enriched among mRNAs with Changed Translational Efficiency, Related to Figure 5.

bioRxiv preprint doi: https://doi.org/10.1101/2024.11.02.621675; this version posted November 3, 2024. The copyright holder for this preprint (which was not certified by peer review) is the author/funder, who has granted bioRxiv a license to display the preprint in perpetuity. It is made available under aCC-BY 4.0 International license.

mRNAs whose translational efficiency increased or decreased in round spermatids compared to secondary spermatocytes (top) or primary spermatocytes (bottom) were examined for enrichment for Gene Ontology terms (biological processes) using Panther database.

Figure S6. GC-2spd(ts) Cells Do Not Express piRNAs And Most piRNA Pathway Genes, Related to Figure 5.

(A) Length profiles for ≥20-nt small RNAs from two biological replicates of GC-2spd(ts) cells. At left, all small RNAs were cloned and sequenced. At right, sodium periodate pre-treatment ensured cloning of only small RNAs with 2′-modified termini (e.g., 2′-*O*-methylated piRNAs).

(B) Mean steady-state abundance of mRNAs encoding piRNA pathway proteins. Data are mean and SD for C57BL/6 primary spermatocytes (n = 3) and GC-2spd(ts) cells (n =2).

(C) Steady-state abundance of SV40-derived transcripts in C57BL/6 primary spermatocytes (n = 2) and GC-2spd(ts) cells (n =2).

bioRxiv preprint doi: https://doi.org/10.1101/2024.11.02.621675; this version posted November 3, 2024. The copyright holder for this preprint (which was not certified by peer review) is the author/funder, who has granted bioRxiv a license to display the preprint in perpetuity. It is made available under aCC-BY 4.0 International license.

SUPPLEMENTARY TABLES

Table S1. Mouse strains used in this study.

Table S2. Change in transcript steady-state abundance in *pi9*$^{-/-}$, *pi17*$^{-/-}$, and *pi9*$^{-/-}$*pi17*$^{-/-}$ primary spermatocytes vs C57BL/6.

Table S3. Known or proposed molecular function for genes whose abundance changes in *pi9*$^{-/-}$ or *pi17*$^{-/-}$ primary spermatocytes vs C57BL/6.

Table S4. Steady-state abundance of pachytene piRNAs in C57BL/6 primary spermatocytes.

Table S5. Identifiable cleavage targets of pachytene piRNAs in primary spermatocytes.

(A) Identifiable cleavage targets of *pi9*, *pi17* piRNAs and piRNAs whose biogenesis is initiated by *pi9* or *pi17* piRNAs in primary spermatocytes.

(B) Identifiable cleavage targets of piRNAs whose abundance does not change in *pi9*$^{-/-}$*pi17*$^{-/-}$ primary spermatocytes (control piRNAs).

Table S6. Genes whose translational efficiency changes in C57BL/6 round spermatids vs secondary or primary spermatocytes.

Data are for all RNAs with ≥10 TPM of ribosome occupancy in C57BL/6 in primary .

Table S7. Change in steady-state abundance of poly(A)+ RNAs and ribosome footprints in *pi9*$^{-/-}$*pi17*$^{-/-}$ vs C57BL/6.

Data are for primary spermatocytes (A), primary spermatocytes (B), and round spermatids (C).

bioRxiv preprint doi: https://doi.org/10.1101/2024.11.02.621675; this version posted November 3, 2024. The copyright holder for this preprint (which was not certified by peer review) is the author/funder, who has granted bioRxiv a license to display the preprint in perpetuity. It is made available under aCC-BY 4.0 International license.

Table S8. Sequence of *Trp53* mRNA assembled de novo using RNA-seq data from GC-2spd(ts) cells.

Table S1. Mouse strains used in this study.

Strain	Two guide RNAs sequences	Deletion coordinates (mm10)	Genotyping mutant allele		Genotyping wild-type allele	
			Primers	Amplicon size	Primers	Amplicon size
pi2$^{-/-}$ (*pi2*$^{em1Pdz/ em1Pdz}$)	GCT TGA TCG TCA GGG ACT AA<br>TCA GAG GCT AAG TCC CAT TA	chr2:92539403–92542146	CCA CCT CCA GCT CTT CCT CT<br>TTA GCT GCC TCA AGA GTG GC	Mut 732 bp	CCC TTG ATC ATA CCC ACC TCC<br>TGT CAA CAA ACC CCC AGG AC	501 bp
pi6$^{-/-}$ (*pi6*$^{em1Pdz/ em1Pdz}$) reported in Wu et al., *Nat Genet* 2020; MGI 6441985	GAC TGC CTA CTC CAA GAT AG<br>CAC ACA AGT GCC CAA CGA AA	chr6:127796350-127796474	ATC CTC CCA GAT GGC TCT GT<br>TGC CCA CTT TAC TGA GGC TG	WT 984 bp<br>Mut 767 bp	GGC CAC TGG CAG TTA GTT CT<br>TGC CCA CTT TAC TGA GGC TG	396 bp
pi6$^{-/-}$ (*pi6*$^{em2Pdz/ em2Pdz}$) reported in Wu et al., *Nat Genet* 2020; MGI 6441998	ACG GTG GGT TCT ATC CAA TG<br>GGA TAG AGT AAG TGA GAA GC	chr6:127796350-127796474	ATC CTC CCA GAT GGC TCT GT<br>TGC CCA CTT TAC TGA GGC TG	WT 984 bp<br>Mut 859 bp	GGC CAC TGG CAG TTA GTT CT<br>TGC CCA CTT TAC TGA GGC TG	396 bp
pi7$^{-/-}$ (*pi7*$^{em1Pdz/ em1Pdz}$)	CCG GGG CCT GCA AAG AAG AA<br>GAC CAC CCT GAA ACC TGT AA	chr7:73816369-73816663	CAT GT CGT TGC TGG GCA AAA<br>GTG GAC CTG TTG CAG GAA CT	WT 983 bp<br>Mut 664 bp	CCC TTT GCC TAG GAC TGT GG<br>CAT GTC GTT GCTG GGC AAA A	488 bp
pi7$^{-/-}$ (*pi7*$^{em2Pdz/ em2Pdz}$)	ACA GGG ATA ATA GCT ATC CC<br>CAC TAG GAT TCC CGT ATC AG	chr7:73816243-73816716	CAT GT CGT TGC TGG GCA AAA<br>GTG GAC CTG TTG CAG GAA CT	WT 983 bp<br>Mut 510 bp	CCC TTT GCC TAG GAC TGT GG<br>CAT GTC GTT GCTG GGC AAA A	488 bp
pi9$^{-/-}$ (*pi9*$^{em1Pdz/ em1Pdz}$)	GGC CTG CAG CAT GCT CTT GC<br>GTT TAG GGT TTG GGT AAG TT	chr9:67733702–67734069	AGA TCC AGA GGC AGG CTT TT<br>TGC CAG CT CTC TTG TCA GAA	WT 774 bp<br>Mut 393 bp	CGT GGA CAA CAG GGA CAC TA<br>CCA CCC CAA ATG CCA TGA AG	307 bp

pi9$^{-/-}$ (*pi9*$^{em2Pdz/ em2Pdz}$)	AGA GTA CGA GGC TAT ACG GA<br><br>AAT AAT CCC ACG GAC TCA CC	chr9:67733639-67734194	AGA TCC AGA GGC AGG CTT TT<br><br>TGC CAG CT CTC TTG TCA GAA	WT 774 bp<br>Mut 219 bp	CGT GGA CAA CAG GGA CAC TA<br><br>CCA CCC CAA ATG CCA TGA AG	307 bp
pi17$^{-/-}$ (*pi17*$^{em1Pdz/ em1Pdz}$) reported in Wu et al., *Nat Genet* 2020; MGI 6441981	GTC CCT TCA CAC GGC CGT TTA<br><br>GCT CTG TCT GAC AAC GGG AC	chr17:27324887–27325439	CGC AGC CCA TCC ATT TCT TG<br><br>GAC TAG CGC CAG TTT CCA CT	WT 1000 bp<br>Mut 448 bp	AGG TCT GCA CGT AGT CTC CT<br><br>GGG TGT GGC CAC ATG TAT CA	368 bp
pi17$^{-/-}$ (*pi17*$^{em2Pdz/ em2Pdz}$) MGI 6441982	ACC GCT GCG CGC CGT GGG AC<br><br>CTG GGA ATC CGG GGT AGC GG	chr17:27324971-27325488	CGC AGC CCA TCC ATT TCT TG<br><br>GAC TAG CGC CAG TTT CCA CT	WT 1000 bp<br>Mut 483 bp	AGG TCT GCA CGT AGT CTC CT<br><br>GGG TGT GGC CAC ATG TAT CA	368 bp

bioRxiv preprint doi: https://doi.org/10.1101/2024.11.02.621675; this version posted November 3, 2024. The copyright holder for this preprint (which was not certified by peer review) is the author/funder, who has granted bioRxiv a license to display the preprint in perpetuity. It is made available under aCC-BY 4.0 International license.

Table S3A. Molecular function of genes whose abundance changes significantly in *pi9*$^{-/-}$ primary spermatocytes (FDR<0.01).

Gene (*-pi9 target)	Fold change *pi9*$^{-/-}$/ *C57BL/6*	Molecular function	References
Aen*	2.4	ssDNA and dsDNA exonuclease, induces apoptosis	(Kawase et al., 2008)
Zbtb26*	2.1	Transcription factor	(Mance et al., 2024)
Champ1*	1.8	Regulator of homologous recombination in DNA damage response	(Li et al., 2022)
Brca2*	1.7	Directs RAD51 to ssDNA during DNA damage response	(Holloman, 2011)
Mrpl27	1.6	Nuclear encoded mitochondrial ribosomal protein	(Gruschke et al., 2010)
Gzf1*	1.5	Transcription factor; implicated in regulation of cell proliferation	(Morinaga et al., 2005; Dambara et al., 2007)
Idh1	0.4	Isocitrate dehydrogenase (cytoplasmic)	(Pirozzi and Yan, 2021)

Table S3B. Molecular function of genes whose abundance changes significantly in *pi17*$^{-/-}$ primary spermatocytes (FDR<0.01).

Gene (*-pi17 target)	Fold change *pi17*$^{-/-}$/ *C57BL/6*	Molecular function	References
Slc41a1*	4.4	Magnesium transporter	(Schäffers et al., 2018; Ilenwabor et al., 2022)
Paqr8*	2.1	Member of progestin and adipoQ receptor (PAQR) protein family; required for tumor survival	(Chen et al., 2023; Pilon and Ruiz, 2023)
Urgcp*	2.0	Upregulator Of Cell Proliferation; contains very large inducible GTPase (VLIG)-type guanine nucleotide-binding domain; implicated in regulation of cell proliferation	(Xie et al., 2012; Xing et al., 2015)
Ywhaz*	2.0	Member of 14-3-3 protein family that regulate signaling pathways; implicated in control of cell proliferation	(Li et al., 2010; Nishimura et al., 2013)
Zfp473*	1.9	Zinc-finger protein	
Acsl3*	1.8	Long-chain acyl-coenzyme A synthase; implicated in regulation of cell proliferation	(Sebastiano et al., 2020; Saliakoura et al., 2020)
Gm11635	1.7	121-aa protein with 25% identity to *Pongo pygmaeus* BRCA1 amino acid residues 899–1022	
Zdhhc16*	1.7	Zinc-finger containing palmitoyltransferase; implicated in regulation of cell proliferation	(Sun et al., 2022)
Chp1*	1.5	Regulator of endoplasmic reticulum glycerolipid synthesis	(Zhu et al., 2019)
Tktl2	1.5	Member of transketolase protein family	(Deshpande et al., 2019)
Cox7a2l*	1.4	Regulator of mitochondrial respirasome biogenesis; implicated in regulation of cell proliferation	(Lobo-Jarne et al., 2018; Ikeda et al., 2019)

bioRxiv preprint doi: https://doi.org/10.1101/2024.11.02.621675; this version posted November 3, 2024. The copyright holder for this preprint (which was not certified by peer review) is the author/funder, who has granted bioRxiv a license to display the preprint in perpetuity. It is made available under aCC-BY 4.0 International license.

References from Table S3.

Chen, Saisai, et al. (2023), 'PAQR8 promotes breast cancer recurrence and confers resistance to multiple therapies', *Breast Cancer Research*, 25 (1), 1.

Dambara, A, et al. (2007), 'Nucleolin modulates the subcellular localization of GDNF-inducible zinc finger protein 1 and its roles in transcription and cell proliferation.', *Exp Cell Res*, 313 (17), 3755-66.

Deshpande, GP, HG Patterton, and M Faadiel Essop (2019), 'The human transketolase-like proteins TKTL1 and TKTL2 are bona fide transketolases.', *BMC Struct Biol*, 19 (1), 2.

Gruschke, S, et al. (2010), 'Proteins at the polypeptide tunnel exit of the yeast mitochondrial ribosome.', *J Biol Chem*, 285 (25), 19022-28.

Holloman, William K (2011), 'Unraveling the mechanism of BRCA2 in homologous recombination', *Nature structural & molecular biology*, 18 (7), 748-54.

Ikeda, K, et al. (2019), 'Mitochondrial supercomplex assembly promotes breast and endometrial tumorigenesis by metabolic alterations and enhanced hypoxia tolerance.', *Nat Commun*, 10 (1), 4108.

Ilenwabor, BP, et al. (2022), 'SLC41A1 knockout mice display normal magnesium homeostasis.', *Am J Physiol Renal Physiol*, 323 (5), F553-63.

Kawase, Tatsuya, et al. (2008), 'p53 target gene AEN is a nuclear exonuclease required for p53-dependent apoptosis', *Oncogene*, 27 (27), 3797-810.

Li, Feng, et al. (2022), 'CHAMP1 binds to REV7/FANCV and promotes homologous recombination repair', *Cell reports*, 40 (9),

Li, Y, et al. (2010), 'Amplification of LAPTM4B and YWHAZ contributes to chemotherapy resistance and recurrence of breast cancer.', *Nat Med*, 16 (2), 214-18.

Lobo-Jarne, T, et al. (2018), 'Human COX7A2L Regulates Complex III Biogenesis and Promotes Supercomplex Organization Remodeling without Affecting Mitochondrial Bioenergetics.', *Cell Rep*, 25 (7), 1786-1799.e4.

Mance, L, et al. (2024), 'Dynamic BTB-domain filaments promote clustering of ZBTB proteins.', *Mol Cell*, 84 (13), 2490-2510.e9.

Morinaga, T, et al. (2005), 'GDNF-inducible zinc finger protein 1 is a sequence-specific transcriptional repressor that binds to the HOXA10 gene regulatory region.', *Nucleic Acids Res*, 33 (13), 4191-201.

Nishimura, Y, et al. (2013), 'Overexpression of YWHAZ relates to tumor cell proliferation and malignant outcome of gastric carcinoma', *British journal of cancer*, 108 (6), 1324-31.

Pilon, M and M Ruiz (2023), 'PAQR proteins and the evolution of a superpower: Eating all kinds of fats: Animals rely on evolutionarily conserved membrane homeostasis proteins to compensate for dietary variation.', *Bioessays*, 45 (9), e2300079.

Pirozzi, CJ and H Yan (2021), 'The implications of IDH mutations for cancer development and therapy.', *Nat Rev Clin Oncol*, 18 (10), 645-61.

Saliakoura, Maria, et al. (2020), 'The ACSL3-LPIAT1 signaling drives prostaglandin synthesis in non-small cell lung cancer', *Oncogene*, 39 (14), 2948-60.

Schäffers, OJM, et al. (2018), 'The rise and fall of novel renal magnesium transporters.', *Am J Physiol Renal Physiol*, 314 (6), F1027-33.

Sebastiano, M Rossi, C Pozzato, and M Saliakoura… (2020), 'ACSL3–PAI-1 signaling axis mediates tumor-stroma cross-talk promoting pancreatic cancer progression', *Science …*,

Sun, Y, et al. (2022), 'S-palmitoylation of PCSK9 induces sorafenib resistance in liver cancer by activating the PI3K/AKT pathway.', *Cell Rep*, 40 (7), 111194.

Xie, C, et al. (2012), 'Upregulator of cell proliferation predicts poor prognosis in hepatocellular carcinoma and contributes to hepatocarcinogenesis by downregulating FOXO3a.', *PLoS One*, 7 (7), e40607.

Xing, Sizhong, et al. (2015), 'URG4/URGCP enhances the angiogenic capacity of human hepatocellular carcinoma cells in vitro via activation of the NF-κB signaling pathway', *BMC cancer*, 15 1-12.

Zhu, XG, et al. (2019), 'CHP1 Regulates Compartmentalized Glycerolipid Synthesis by Activating GPAT4.', *Mol Cell*, 74 (1), 45-58.e7.

Table S8. Sequence of *Trp53* mRNA assembled de novo with raw RNA-seq reads from GC-2spd(ts) cells using Trinity. Mutation that causes Ala-to-Val substitution is highlighted in red.

Rep1	AAUAAAAGAGCCCACAACCCCUCACUCGGCGCGCCAGUCCUCCGAUUGACUGAGUCGCCCGAUCUCCCGCACCUAGCAUUCAGG<br>CCCUCAUCCUCCUCCUUCCCAGCAGGGUGUCACGCUUCUCCGAAGACUGGAUGACUGCCAUGGAGGAGUCACAGUCGGAUAUC<br>AGCCUCGAGCUCCCUCUGAGCCAGGAGACAUUUUCAGGCUUAUGGAAACUACUUCCUCCAGAAGAUAUCCUGCCAUCACCUCAC<br>UGCAUGGACGAUCUGUUGCUGCCCCAGGAUGUUGAGGAGUUUUUUGAAGGCCCAAGUGAAGCCCUCCGAGUGUCAGGAGCUCC<br>UGCAGCACAGGACCCUGUCACCGAGACCCCUGGGCCAGUGGCCCCUGCCCCAGCCACUCCAUGGCCCCUGUCAUCUUUUGUCC<br>CUUCUCAAAAAACUUACCAGGGCAACUAUGGCUUCCACCUGGGCUUCCUGCAGUCUGGGACAGCCAAGUCUGUUAUGUGCACGU<br>ACUCUCCUCCCCUCAAUAAGCUAUUCUGCCAGCUGGUGAAGACGUGCCCUGUGCAGUUGUGGGUCAGCGCCACACCUCCAGCU<br>GGGAGCCGUGUCCGCGCCAUGGCCAUCUACAAGAAGUCACAGCACAUGACGGAGGUCGUGAGACGCUGCCCCCACCAUGAGCG<br>CUGCUCCGAUGGUGAUGGCCUGGCUCCUCCCCAGCAUCUUAUCCGGGUGGAAGGAAAUUUGUAUCCCGAGUAUCUGGAAGACA<br>GGCAGACUUUUCGCCACAGCGUGGUGGUACCUUAUGAGCCACCCGAGGCCGGCUCUGAGUAUACCACCAUCCACUACAAGUACA<br>UGUGUAAUAGCUCCUGCAUGGGGGGCAUGAACCGCCGACCUAUCCUUACCAUCAUCACACUGGAAGACUCCAGUGGGAACCUUC<br>UGGGACGGGACAGCUUUGAGGUUCGUGUUUGUGCCUGCCCUGGGAGAGACCGCCGUACAGAAGAAGAAAAUUUCCGCAAAAAGG<br>AAGUCCUUUGCCCUGAACUGCCCCCAGGGAGCGCAAAGAGAGCGCUGCCCACCUGCACAAGCGCCUCUCCCCCGCAAAAGAAAA<br>AACCACUUGAUGGAGAGUAUUUCACCCUCAAGAUCCGCGGGCGUAAACGCUUCGAGAUGUUCCGGGAGCUGAAUGAGGCCUUAG<br>AGUUAAAGGAUGCCCAUGCUACAGAGGAGUCUGGAGACAGCAGGGCUCACUCCAGCUACCUGAAGACCAAGAAGGGCCAGUCUA<br>CUUCCCGCCAUAAAAAAACAAUGGUCAAGAAAGUGGGGCCUGACUCAGACUGACUGCCUCUGCAUCCCGCCCCCCCCCCCUGC<br>AGCAAUGGCAACAACGUUGCGCAAAC
Rep2	AUUGGGACCAUCCUGGCUGUAGGUAGCGACUACAGUUAGGGGGCACCUAGCAUUCAGGCCCUCAUCCUCCUCCUUCCCAGCAG<br>GGUGUCACGCUUCUCCGAAGACUGGAUGACUGCCAUGGAGGAGUCACAGUCGGAUAUCAGCCUCGAGCUCCCUCUGAGCCAGG<br>AGACAUUUUCAGGCUUAUGGAAACUACUUCCUCCAGAAGAUAUCCUGCCAUCACCUCACUGCAUGGACGAUCUGUUGCUGCCCC<br>AGGAUGUUGAGGAGUUUUUUGAAGGCCCAAGUGAAGCCCUCCGAGUGUCAGGAGCUCCUGCAGCACAGGACCCUGUCACCGAG<br>ACCCCUGGGCCAGUGGCCCCUGCCCCAGCCACUCCAUGGCCCCUGUCAUCUUUUGUCCCUUCUCAAAAAACUUACCAGGGCAAC<br>UAUGGCUUCCACCUGGGCUUCCUGCAGUCUGGGACAGCCAAGUCUGUUAUGUGCACGUACUCUCCUCCCCUCAAUAAGCUAUU<br>CUGCCAGCUGGUGAAGACGUGCCCUGUGCAGUUGUGGGUCAGCGCCACACCUCCAGCUGGGAGCCGUGUCCGCGCCAUGGCCA<br>UCUACAAGAAGUCACAGCACAUGACGGAGGUCGUGAGACGCUGCCCCCACCAUGAGCGCUGCUCCGAUGGUGAUGGCCUGGCU<br>CCUCCCCAGCAUCUUAUCCGGGUGGAAGGAAAUUUGUAUCCCGAGUAUCUGGAAGACAGGCAGACUUUUCGCCACAGCGUGGUG<br>GUACCUUAUGAGCCACCCGAGGCCGGCUCUGAGUAUACCACCAUCCACUACAAGUACAUGUGUAAUAGCUCCUGCAUGGGGGGC<br>AUGAACCGCCGACCUAUCCUUACCAUCAUCACACUGGAAGACUCCAGUGGGAACCUUCUGGGACGGGACAGCUUUGAGGUUCGU<br>GUUUGUGCCUGCCCUGGGAGAGACCGCCGUACAGAAGAAGAAAAUUUCCGCAAAAAGGAAGUCCUUUGCCCUGAACUGCCCCCA<br>GGGAGCGCAAAGAGAGCGCUGCCCACCUGCACAAGCGCCUCUCCCCCGCAAAAGAAAAAACCACUUGAUGGAGAGUAUUUCACC<br>CUCAAGAUCCGCGGGCGUAAACGCUUCGAGAUGUUCCGGGAGCUGAAUGAGGCCUUAGAGUUAAAGGAUGCCCAUGCUACAGAG<br>GAGUCUGGAGACAGCAGGGCUCACUCCAGCUACCUGAAGACCAAGAAGGGCCAGUCUACUUCCCGCCAUAAAAAAACAAUGGUCA<br>AGAAAGUGGGGCCUGACUCAGACUGACUGCCUCUGCGGGGGGGGGGGGGGGGGGGGGGG

Table S9. Sequences of oligonucleotides used in this study

	Name	Sequence (5′-to-3′)	Notes
Adapters, oligos, and primers for small RNA sequencing and RFP (Ribosome Footprint Profiling)	Equimolar mix of nine spike-in RNA oligonucleotides, only used for small RNA sequencing	/phos/UGCUAGUCUGUUAUCGACCUGACCUCAUAG	5′ monophosphorylated RNA
		/phos/UGCUAGUCUGUUCGAUACCUGACCUCAUAG	
		/phos/UGCUAGUCUGUUGUCACGAAGACCUCAUAG	
		/phos/UGCUAGUCUUAUCGACCUCCUCAUAG	
		/phos/UGCUAGUCUUCGAUACCUCCUCAUAG	
		/phos/UGCUAGUCUUGUCACGAACCUCAUAG	
		/phos/UGCUAGUUAUCGACCUUCAUAG	
		/phos/UGCUAGUUCGAUACCUUCAUAG	
		/phos/UGCUAGUUGUCACGAAUCAUAG	
	3′ DNA adapter	/rApp/NNNGTCNNNTAGNNNTGGAATTCTCGGGTGCCAAGG/ddC/	5′ adenylated, 3′ dideoxycytosine blocked DNA adapter
	Equimolar mix of two 5′ RNA adaptors	GUUCAGAGUUCUACAGUCCGACGAUCNNNCGANNNUACNNN	RNA
		GUUCAGAGUUCUACAGUCCGACGAUCNNNAUCNNNAGUNNN	
	RT primer	CCTTGGCACCCGAGAATTCCA	DNA
	Forward (P5) primer	AATGATACGGCGACCACCGAGATCTACACGTTC AGAGTTCTACAGTCCGA	DNA primers for final amplification of library, XXXXXX represents 6-nt sequencing barcode
	Reverse (P7) primer	CAAGCAGAAGACGGCATACGAGATXXXXXX<br>GTGACTGGAGTTCCTTGGCACCCGAGAATTCCA	
Three sets of unique molecular identifier (UMI) containing RNAseq adapters	Adapter set 1	/phos/CCCNNNNNNAGATCGGAAGAGCACACGTCT	Two adapters in each set are first annealed to each other in three separate tubes, then the three annealed sets are equimolarly mixed to a final total concentration of 3.3 µM each duplex
		ACACTCTTTCCCTACACGACGCTCTTCCGATCTNNNNNNGGGT	
	Adapter set 2	/phos/GATNNNNNNAGATCGGAAGAGCACACGTCT	
		ACACTCTTTCCCTACACGACGCTCTTCCGATCTNNNNNNATCT	
	Adapter set 3	/phos/TGANNNNNNAGATCGGAAGAGCACACGTCT	
		ACACTCTTTCCCTACACGACGCTCTTCCGATCTNNNNNNTCAT	
Adapters and primers for cloning and sequencing long 5′ mono-phosphorylated RNAs	Equimolar mix of two 5′ RNA adaptors	GUUCAGAGUUCUACAGUCCGACGAUCNNNCGANNNUACNNN	RNA
		GUUCAGAGUUCUACAGUCCGACGAUCNNNAUCNNNAGUNNN	
	RT primer	GCACCCGAGAATTCCANNNNNNNNN	DNA
	PCR 1 forward primer	CTACACGTTCAGAGTTCTACAGTCCGA	DNA primers for the first PCR amplification

	PCR 1 reverse primer	GCCTTGGCACCCGAGAATTCCA	
	Forward (P5) primer	AATGATACGGCGACCACCGAGATCTACACGTTC AGAGTTCTACAGTCCGA	DNA primers for the second PCR amplification
	Reverse (P7) primer	CAAGCAGAAGACGGCATACGAGATXXXXXX GTGACTGGAGTTCCTTGGCACCCGAGAATTCCA	

bioRxiv preprint doi: https://doi.org/10.1101/2024.11.02.621675; this version posted November 3, 2024. The copyright holder for this preprint (which was not certified by peer review) is the author/funder, who has granted bioRxiv a license to display the preprint in perpetuity. It is made available under aCC-BY 4.0 International license.

Table S10. Number of Primary Spermatocytes and GC-2spd(ts) cells and Amount of Spike-In Mix Used to Prepare Small RNA Sequencing Libraries.

Genotype		Trial	Cell number	Amount of spike-in, attomol
C57BL/6 data are from Gainetdinov et al, *Nature* 2023	SRR21528503	Rep1	31400	370
	SRR21528502	Rep2	68900	4000
	SRR21528501	Rep3	47200	3000
	SRR21528500	Rep4	89100	4000
	SRR21528499	Rep5	48900	3000
	SRR21528498	Rep6	60300	4000
	SRR21528497	Rep7	63100	4000
	SRR21528495	Rep8	99700	4000
	SRR21528494	Rep9	112500	4000
	SRR21528493	Rep10	116400	4000
	SRR21528492	Rep11	137000	4000
	SRR21528491	Rep12	112000	4000
pi9$^{-/-}$		Rep1	41,600	3,000
		Rep2	47,100	3,000
		Rep3	45,600	3,000
		Rep4	59,600	4,000
		Rep5	71,200	4,000
		Rep6	95,800	4,000
		Rep7	56,000	4,000
pi17$^{-/-}$		Rep1	59,900	4,000
		Rep2	36,460	4,000
		Rep3	76,570	4,000
		Rep4	54,000	4,000
		Rep5	153,300	4,000
		Rep6	91,300	4,000
pi9$^{-/-}$*pi17*$^{-/-}$		Rep1	40,000	4,000
		Rep2	36,000	4,000
		Rep3	33,500	4,000
pi6$^{-/-}$		Rep1	45,400	4,000
GC-2spd(ts)		Rep1	100,000	400
		Rep2	100,000	400
		Rep3	100,000	400
		Rep4	100,000	400

All Supplemental Figures
bioRxiv preprint doi: https://doi.org/10.1101/2024.11.02.621675; this version posted November 3, 2024. The copyright holder for this preprint (which was not certified by peer review) is the author/funder, who has granted bioRxiv a license to display the preprint in perpetuity. It is made available under aCC-BY 4.0 International license.
Click here to access/download;Supplemental Figure;Cecchini et al. All Supplemental Figures.pdf

Cecchini et al., Figure S1

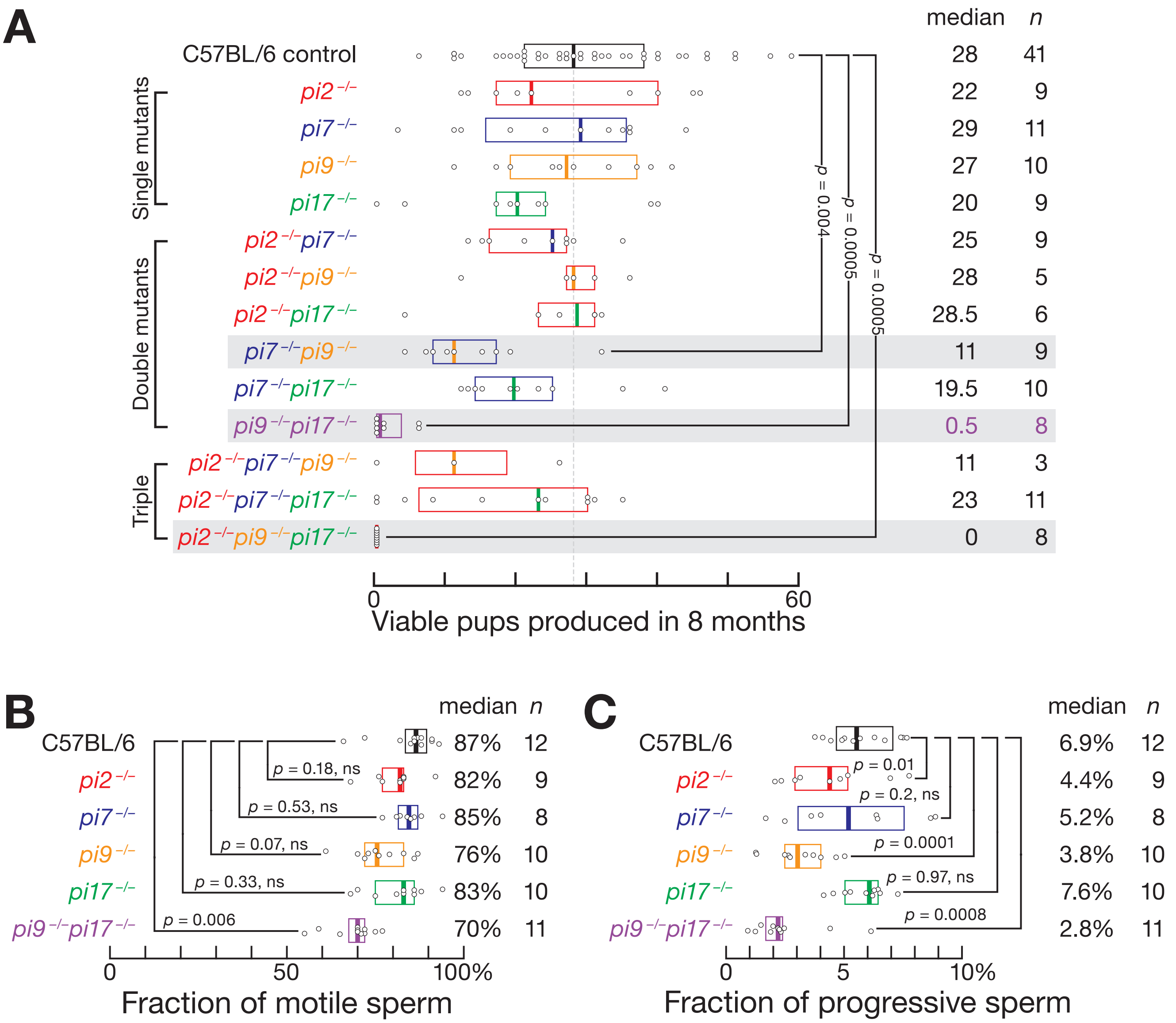

D

	Sperm donor genotype	Trial	Oocytes	Two-cell embryos	Mean ± SD
Zona intact	C57BL/6	1	176	143 (81%)	
		2	223	214 (96%)	70 ± 30%
		3	384	141 (37%)	
	pi9−/−*pi17*−/−	1	111	0 (0%)	
		2	166	31 (19%)	10 ± 10%
		3	426	8 (2%)	
Zona removed	C57BL/6	1	83	31 (37%)	
		2	86	85 (99%)	80 ± 40%
		3	299	290 (97%)	
	pi9−/−*pi17*−/−	1	75	40 (53%)	
		2	66	64 (97%)	80 ± 30%
		3	297	285 (96%)	

A

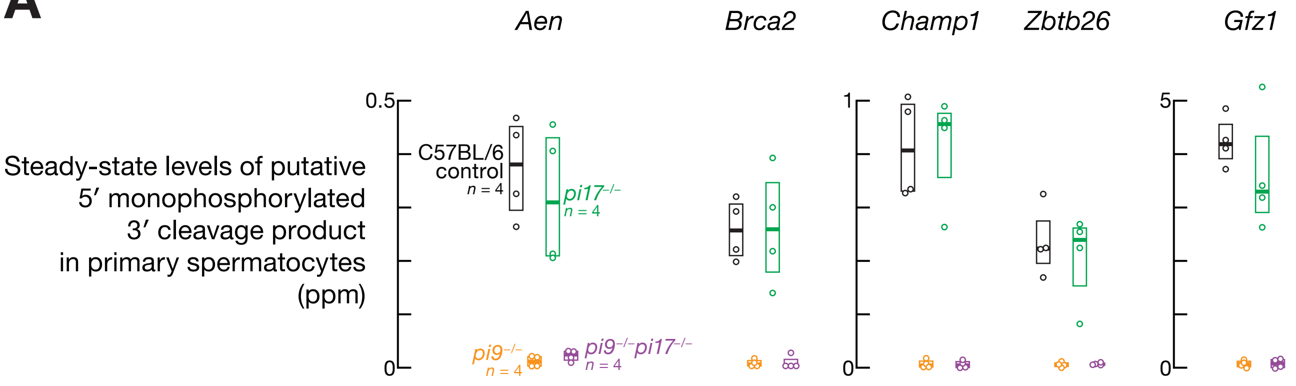

	Kruskal-Wallis test *p*-value	Benjamini-Hochberg corrected, pairwise Mann-Whitney test *p*-value for C57BL/6 versus:		
		$pi9^{-/-}$	$pi17^{-/-}$	$pi9^{-/-}pi17^{-/-}$
Aen	0.008	0.043	0.49	0.043
Brca2	0.009	0.044	1	0.044
Champ1	0.01	0.043	0.88	0.043
Zbtb26	0.01	0.043	1	0.043
Gzf1	0.009	0.043	0.41	0.043

B

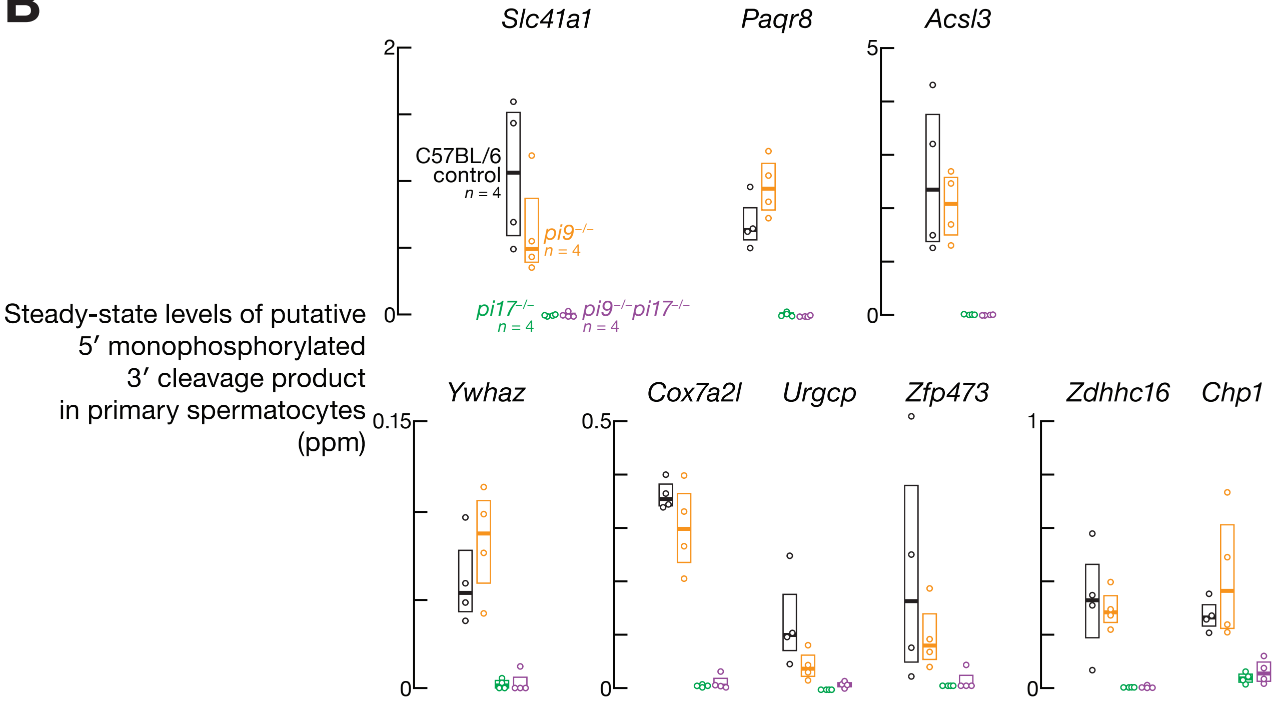

	Kruskal-Wallis test *p*-value	Benjamini-Hochberg corrected, pairwise Mann-Whitney test *p*-value for C57BL/6 versus:		
		$pi9^{-/-}$	$pi17^{-/-}$	$pi9^{-/-}pi17^{-/-}$
Slc41a1	0.008	0.24	0.044	0.044
Paqr8	0.005	0.11	0.043	0.043
Acsl3	0.007	0.89	0.044	0.044
Ywhaz	0.007	0.41	0.044	0.044
Cox7a2l	0.008	0.24	0.043	0.043
Urgcp	0.003	0.06	0.034	0.034
Zfp473	0.007	0.89	0.044	0.044
Zdhhc16	0.011	0.69	0.043	0.043
Chp1	0.009	0.69	0.043	0.043

Cecchini et al., Figure S3

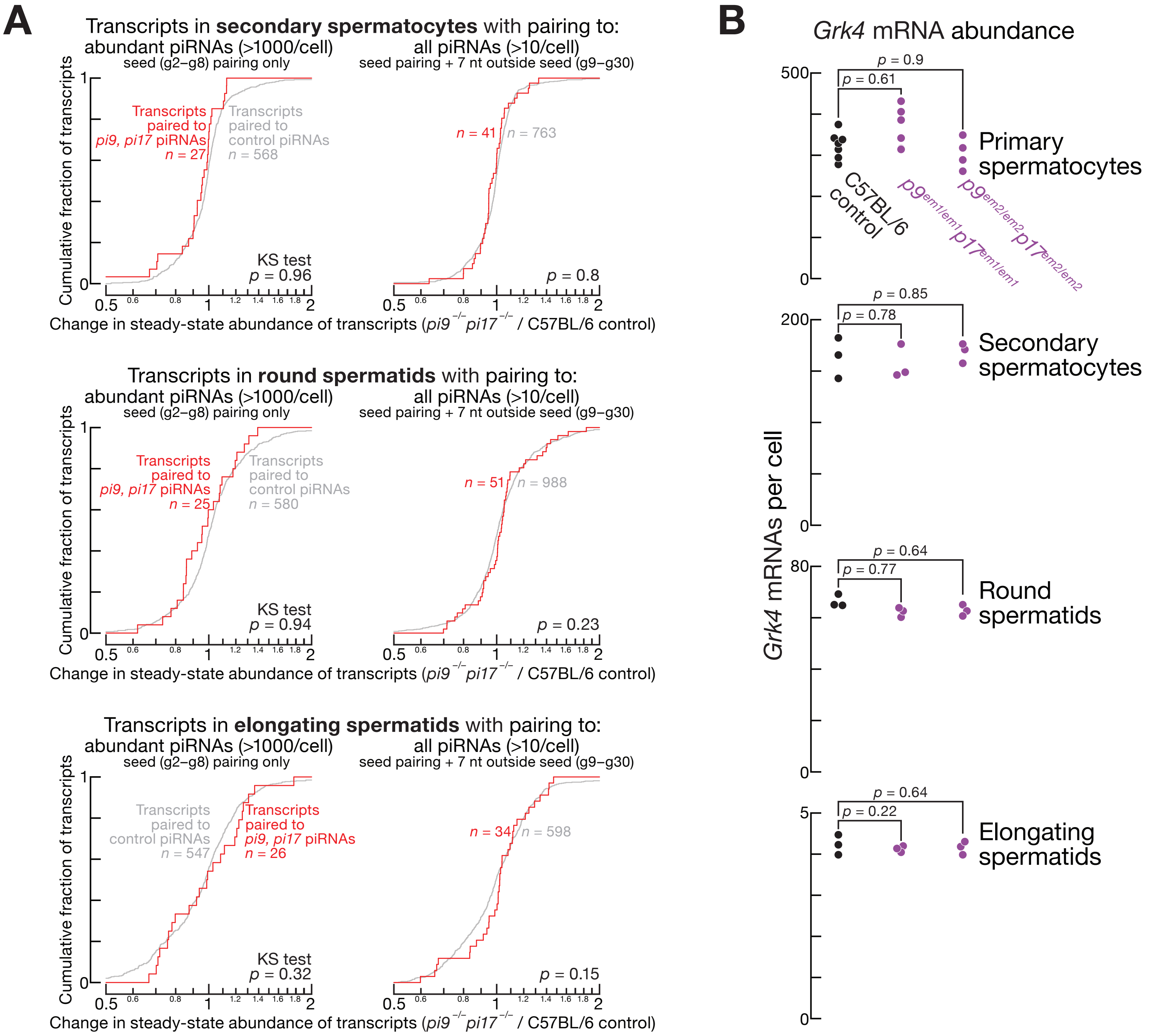

Cecchini et al., Figure S4

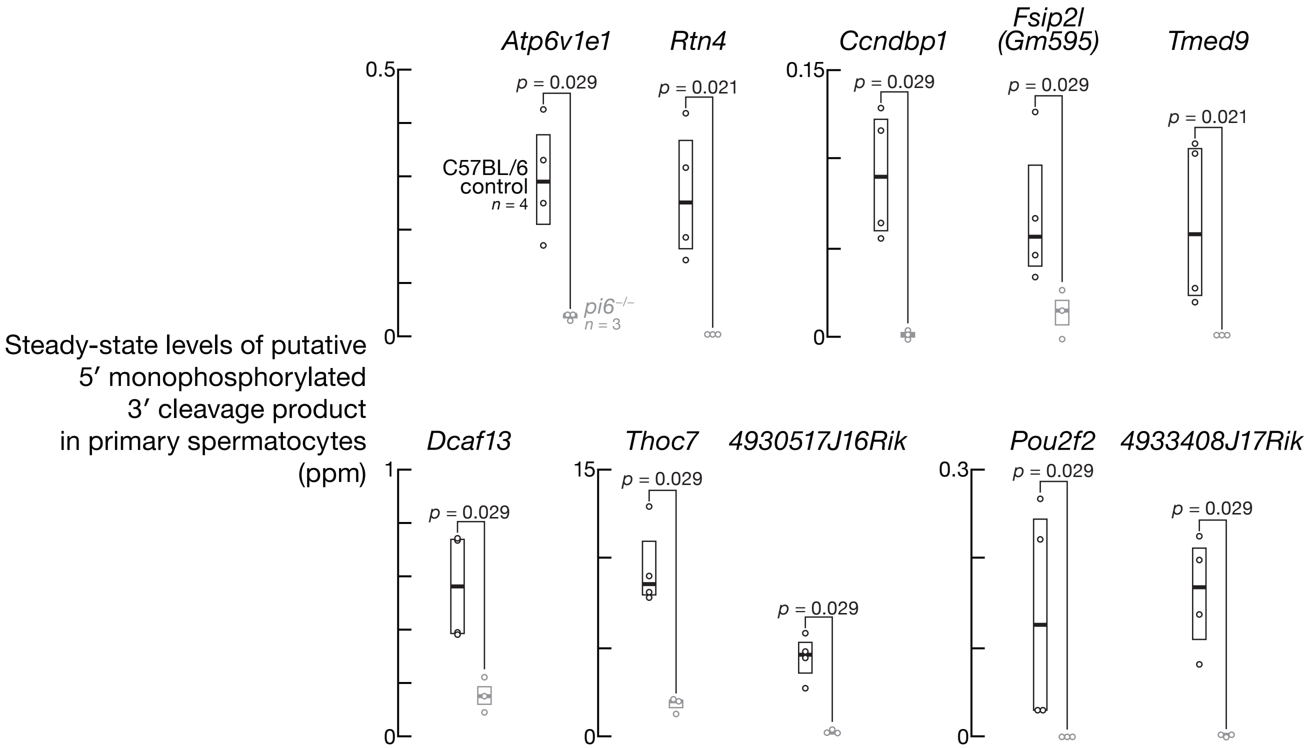

Comparison	Translational efficiency	Gene Ontology term (biological process)	Genes observed	Genes expected	Enrichment	Adjusted *p*-value
Round spermatids vs **secondary spermatocytes**	Translational efficiency **increases ≥2-fold** (89 genes)	binding of sperm to zona pellucida	4	0.19	20.8	2.5×10^{-2}
		flagellated sperm motility	9	0.66	13.7	2.5×10^{-5}
		male gonad development	6	0.60	9.9	2.5×10^{-2}
		spermatid development	8	1.21	6.6	2.3×10^{-2}
	Translational efficiency **decreases ≥1.5-fold** (46 genes)	microtubule cytoskeleton organization	9	1.19	7.5	3.4×10^{-2}

Comparison	Translational efficiency	Gene Ontology term (biological process)	Genes observed	Genes expected	Enrichment	Adjusted *p*-value
Round spermatids vs **primary spermatocytes**	Translational efficiency **increases ≥2-fold** (89 genes)	sperm mitochondrial sheath assembly	4	0.06	66.7	9.4×10^{-5}
		binding of sperm to zona pellucida	5	0.40	12.4	1.7×10^{-2}
		ATP biosynthetic process	7	0.68	10.3	2.5×10^{-3}
	Translational efficiency **decreases ≥1.5-fold** (46 genes)	CRD-mediated mRNA stabilization	3	0.03	90.9	2.1×10^{-2}
		negative regulation of mRNA	3	0.04	82.7	1.4×10^{-2}
		positive regulation of cytoplasmic translation	3	0.05	60.6	1.7×10^{-2}
		positive regulation of transcription Pol II	4	0.22	18.1	4.4×10^{-2}
		mRNA transport	5	0.38	13.1	3.0×10^{-2}
		nuclear export	5	0.44	11.4	4.5×10^{-2}
		establishment of protein localization to organelle	8	1.15	6.9	1.8×10^{-2}
		spermatogenesis	11	2.23	4.9	1.6×10^{-2}
		macromolecule metabolic process	37	19.23	1.9	1.6×10^{-2}

bioRxiv preprint doi: https://doi.org/10.1101/2024.11.02.621675; this version posted November 3, 2024. The copyright holder for this preprint (which was not certified by peer review) is the author/funder, who has granted bioRxiv a license to display the preprint in perpetuity. It is made available under aCC-BY 4.0 International license.

Cecchini et al., Figure S6

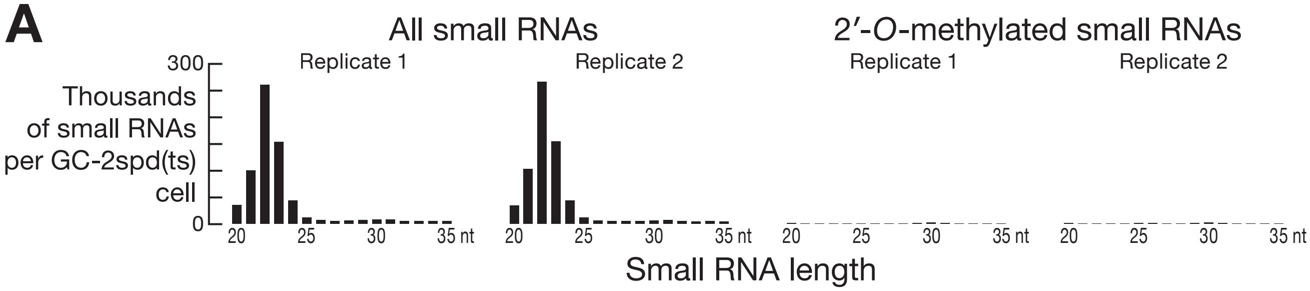

B

Transcript abundance, TPM (transcript per million)

Gene name	C57BL/6 primary spermatocytes, n = 3	GC-2spd(ts) cells, n = 2
Gasz1	11 ± 1	0 ± 0
Ddx4	120 ± 20	0 ± 0
Gpat2	42 ± 5	0 ± 0
Gtsf1	100 ± 10	0 ± 0
Henmt1	37 ± 2	0 ± 0
Mael	440 ± 50	0.4 ± 0.2
Mov10l1	35 ± 4	0 ± 0
Mybl1	23 ± 2	5.5 ± 0.4
Piwil1	260 ± 20	0 ± 0
Piwil2	60 ± 10	0 ± 0
Pld6	100 ± 10	0 ± 0
Pnldc1	6 ± 1	2.3 ± 0.4
Rnf17	32 ± 6	0 ± 0
Tdrd1	72 ± 2	0 ± 0
Tdrd12	43 ± 2	0 ± 0
Tdrd5	55 ± 1	0 ± 0
Tdrd6	350 ± 10	0 ± 0
Tdrd7	120 ± 10	9.0 ± 1
Tdrd9	68 ± 2	0 ± 0
Tdrkh	12 ± 1	2.0 ± 1

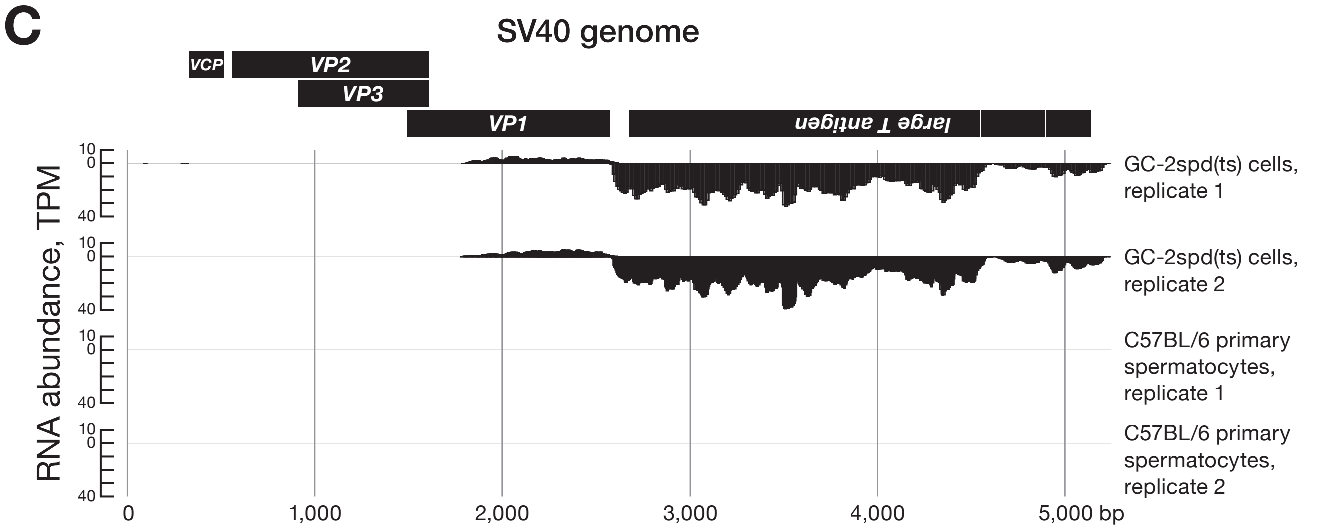

Supplement: Supplement 6 [file NIHPP2024.11.02.621675v1-supplement-6.pdf]
